# Obstructive Coronary Artery Disease and Health Status in Transcatheter Aortic Valve Replacement: A Post Hoc Analysis of the SCOPE I Randomized Clinical Trial

**DOI:** 10.1001/jamanetworkopen.2025.47111

**Published:** 2025-12-09

**Authors:** Daijiro Tomii, Jonas Lanz, Holger Thiele, Dik Heg, Won-Keun Kim, Michael Joner, Helge Möllmann, Christof Burgdorf, Axel Linke, Simon Redwood, Michael Hilker, Lenard Conradi, Sebastian Kerber, Christian Thilo, Stefan Toggweiler, Thomas Walther, Bernard Prendergast, Stephan Windecker, Thomas Pilgrim

**Affiliations:** 1 Department of Cardiology, Bern University Hospital, Inselspital, University of Bern, Bern, Switzerland; 2 Heart Centre Leipzig at Leipzig University, Leipzig, Germany; 3 Department of Clinical Research, University of Bern, Bern, Switzerland; 4 Department of Cardiology, Kerckhoff Heart and Thorax Centre, Bad Nauheim, Germany; 5 German Heart Centre, Technical University of Munich, Munich, Germany; 6 Department of Internal Medicine I, St-Johannes-Hospital, Dortmund, Germany; 7 Heart and Vascular Centre, Bad Bevensen, Germany; 8 Department of Internal Medicine and Cardiology, Heart Centre Dresden, Technische Universität Dresden, Dresden, Germany; 9 Department of Cardiology, St Thomas’ Hospital & Cleveland Clinic London, London, UK; 10 Department of Cardiothoracic Surgery, University Medical Centre, Regensburg, Germany; 11 Department of Cardiovascular Surgery, University Heart Centre Hamburg, Hamburg, Germany; 12 Department of Cardiology, Cardiovascular Centre Bad Neustadt, Bad Neustadt, Germany; 13 Department of Internal Medicine I, RoMed Klinikum Rosenheim, Rosenheim, Germany; 14 Heart Center Lucerne, Luzerner Kantonsspital, Lucerne, Switzerland; 15 Department of Cardiac, Thoracic and Thoracic Vascular Surgery, University Hospital Frankfurt, Frankfurt, Germany

## Abstract

**Importance:**

Aortic stenosis (AS) and obstructive coronary artery disease (CAD) often coexist, yet the impact of obstructive CAD on clinical and patient-reported outcomes in patients undergoing transcatheter aortic valve replacement (TAVR) remains a subject of ongoing debate.

**Objectives:**

To investigate the association of obstructive CAD with clinical outcomes and health status among patients with symptomatic severe AS undergoing TAVR.

**Design, Setting, and Participants:**

This post hoc analysis of the investigator-initiated, multicenter SCOPE I (Safety and Efficacy of the Symetis ACURATE Neo/TF Compared to the Edwards SAPIEN 3 Bioprosthesis for Transcatheter Aortic Valve Implantation by Transfemoral Approach) randomized clinical trial was conducted at 20 tertiary heart centers in Europe from February 8, 2017, to February 2, 2019, with follow-up through 3 years. Data were analyzed from February 17 through August 13, 2025.

**Exposure:**

Presence vs absence of obstructive CAD, defined as greater than 50% stenosis in at least 1 major epicardial coronary vessel.

**Main Outcomes and Measures:**

Parameters of interest included vital and patient-reported disease-specific health status (Kansas City Cardiomyopathy Questionnaire [KCCQ] scores, ranging from 0 to 100, with higher numbers indicating better health status), and clinical efficacy according to Valve Academic Research Consortium (VARC)-3 definitions. Analyses were conducted using the as treated population.

**Results:**

Of 732 patients with symptomatic severe AS undergoing TAVR (mean [SD] age, 82 [4] years; 416 [56.8%] female), obstructive CAD was identified in 373 (51.0%), 144 (38.6%) of whom underwent elective percutaneous coronary intervention (PCI) during the periprocedural period. At 3 years after TAVR, there were no statistically significant differences in patient-reported health status (eg, median [IQR] baseline overall KCCQ scores with CAD, 54.2 [40.3-69.8] vs without CAD, 55.2 [38.5-72.9] and at 3-year follow-up with CAD, 79.7 [64.4-90.6] vs without CAD, 82.3 [68.2-91.7]), mortality (all-cause death: 88 of 373 [24.7%] vs 76 of 359 [22.3%], adjusted hazard ratio, [HR], 0.97 [95% CI, 0.66-1.43]; cardiovascular death: 59 of 373 [17.6%] vs 51 of 359 [15.5%], adjusted HR, 0.87 [95% CI, 0.54-1.42]), and clinical efficacy of TAVR (163 of 313 [52.1%] vs 159 of 298 [53.4%]; adjusted risk ratio, 1.10 [95% CI, 0.92-1.32]) between patients with vs without obstructive CAD. Having (vs not having) obstructive CAD was associated with a numerically albeit not statistically significantly higher risk of myocardial infarction (18 of 372 [5.5%] vs 3 of 359 [1.1%]; adjusted HR, 3.83 [95% CI, 0.96-15.31]). Periprocedural PCI among patients with obstructive CAD did not improve clinical outcomes, patient-reported health status, nor the integrated end points of clinical outcomes and quality of life measures.

**Conclusions and Relevance:**

In this post hoc analysis of SCOPE I, patients with obstructive CAD who underwent TAVR had no statistically significant differences in survival, patient-reported health status, or VARC-3 clinical efficacy compared with patients without CAD through 3 years of follow-up. Findings suggest that a tailored approach may be essential in the treatment of TAVR candidates with severe AS and CAD.

**Trial Registration:**

ClinicalTrials.gov Identifier: NCT03011346

## Key Points

**Question:**

Is the presence of obstructive coronary artery disease (CAD) associated with clinical outcomes and health status among patients with symptomatic severe aortic stenosis (AS) undergoing transcatheter aortic valve replacement (TAVR)?

**Findings:**

In a post hoc analysis of the SCOPE I randomized clinical trial including 732 patients, no statistically significant differences were observed between patients with vs without obstructive CAD in patient-reported health status, mortality, and clinical efficacy according to Valve Academic Research Consortium-3 (VARC-3) definitions of TAVR throughout 3 years of follow-up.

**Meaning:**

These findings suggest that concomitant CAD may not adversely affect survival, patient-reported health status, or VARC-3 clinical efficacy in patients with AS undergoing TAVR.

## Teaser

This secondary post hoc analysis of a randomized clinical trial investigates the association of obstructive coronary artery disease with patient-reported disease-specific health status and clinical efficacy among patients with symptomatic severe aortic stenosis undergoing transcatheter aortic valve replacement.

## Introduction

Aortic stenosis (AS) and obstructive coronary artery disease (CAD) frequently coexist and share common pathophysiological mechanisms that affect prognosis and quality of life.^1,2,3^ In patients undergoing surgical aortic valve replacement, the presence of obstructive CAD is associated with an increased risk of perioperative and late adverse events, whereas the addition of coronary artery bypass grafting in patients with AS and CAD has been associated with improved long-term clinical outcomes and prognosis.^4,5^ However, the clinical impact of obstructive CAD and therapeutic impact of percutaneous coronary intervention (PCI) in patients undergoing transcatheter aortic valve replacement (TAVR) are not fully established.^6,7,8,9^ Among patients with chronic coronary disease, several trials reported similar survival between a default strategy of routine revascularization and a conservative approach directed at optimization of medical therapy followed by revascularization as needed.^10,11^ Nonetheless, revascularization in addition to guideline-directed medical therapy improves symptom relief, quality of life measures, and the risk of spontaneous myocardial infarction.^12,13^ Available evidence in the context of TAVR largely focuses on clinical outcomes, with limited data concerning patient-reported quality of life measures. Since survival alone may not fully capture treatment goals in older patients, integration of clinical outcomes and patient-reported quality of life measures provides a more comprehensive assessment.^14^ In the present study, we aimed to investigate an integrated assessment of clinical outcomes and health-related quality of life measures according to the presence or absence of obstructive CAD in patients undergoing TAVR.

## Methods

### Study Design and Population

The parent study, SCOPE I (Safety and Efficacy of the Symetis ACURATE Neo/TF Compared to the Edwards SAPIEN 3 Bioprosthesis for Transcatheter Aortic Valve Implantation by Transfemoral Approach) was an investigator-initiated, randomized clinical trial conducted at 20 tertiary heart centers across Europe comparing the self-expanding ACURATE Neo (Boston Scientific) and balloon-expandable SAPIEN 3 (Edwards Lifesciences) transcatheter heart valves in older patients with symptomatic severe AS undergoing TAVR. Details of the design and conduct of the trial have been previously reported.^15,16^ In brief, patients aged 75 years or older with symptomatic, severe AS who were deemed to be at increased surgical risk by the Heart Team were randomized in a 1:1 ratio to undergo transfemoral TAVR with an ACURATE Neo or SAPIEN 3 transcatheter heart valve. The trial was approved by the institutional review board or ethics committee at each participating site and was conducted in accordance with the principles of the Declaration of Helsinki.^17^ All patients provided written informed consent before enrollment. In SCOPE I, coronary angiography and coronary computed tomography angiography were included as part of standard preprocedural evaluation, and obstructive CAD was defined as a stenosis higher than 50% in at least 1 major epicardial coronary vessel. Periprocedural PCI was performed at the discretion of the local Heart Team. In this post hoc analysis, patients who underwent TAVR were stratified according to the presence or absence of obstructive CAD, and those with obstructive CAD were further divided into 2 groups according to the use of PCI or conservative management. This study conforms to the Strengthening the Reporting of Observational Studies in Epidemiology (STROBE) reporting guideline.^18^

### Data Collection and Clinical End Points

Follow-up was performed at 30 days and at 1 and 3 years by means of outpatient visits, telephone interviews, and consultation of medical records. Clinical end points were adjudicated by an independent clinical event committee, blinded to treatment allocation, based on Valve Academic Research Consortium-2 (VARC-2) criteria.^15,19^ Patient-reported health status was assessed using the Kansas City Cardiomyopathy Questionnaire (KCCQ)-12 score, encompassing 4 domains^15,20,21^—physical limitation, quality of life, social limitation, and symptom frequency—that were combined to provide an overall summary score. KCCQ scores range from 0 to 100, with higher numbers indicating better health status. For the purpose of the present study, ordinal analysis of overall KCCQ score was applied based on changes from baseline.^14,21^ The VARC-3 definitions recommend reporting both the integrated categorical end point of clinical outcomes and disease-specific health status as well as patient-reported health status. To follow this recommendation, we retrospectively adjudicated the integrated end points based on the adjudicated clinical end points and overall KCCQ score at the 1- and 3-year follow-up visits.^14,21^ A favorable outcome indicated that the patient was alive and that the overall KCCQ score was at least 60, with no reduction in overall KCCQ score of more than10 points from baseline. An acceptable outcome indicated that the patient was alive and that the overall KCCQ score was at least 45, with no reduction in overall KCCQ score of more than 10 points from baseline. An unfavorable outcome indicated that the patient was deceased or that the patient was alive and the overall KCCQ score was lower than 45 or the reduction in the overall KCCQ score was more than 10 points from baseline. Clinical efficacy indicated freedom from the following: all-cause mortality, all stroke, hospitalization for procedure- or valve-related causes, and unfavorable outcome.

### Statistical Analysis

Categorical variables are presented as frequencies and percentages, and differences between groups were evaluated using the χ^2^ test or Fisher exact test for 2 × 2 comparisons. Continuous variables are presented as the mean and SD and compared using *t* tests, given that the central limit theorem states that in general, data will quickly approach a normal distribution as sample sizes increase. Nevertheless, Shapiro-Wilk tests and Q-Q plots were conducted, flagging some variables as potentially nonnormally distributed. For these variables we calculated the Hodges-Lehmann median difference to estimate the difference in KCCQ scores between groups and groups compared using the Mann-Whitney test. Patients who were alive without information on KCCQ score at baseline or follow-up were excluded from the analysis of clinical efficacy (n = 85 and 121 at 1 and 3 years, respectively), whereas patients who were deceased remained in the denominator throughout. Cumulative incidence curves were constructed using the Kaplan-Meier method for time-to-event data, censoring patients at the time of last valid contact (or after death for those who were deceased). Cox proportional hazards models were used to calculate hazard ratios (HRs) and 95% CIs, and risk ratios (RRs) with 95% CIs provided using Poisson regressions for VARC-3 integrated end points. Multivariable adjustment was performed using prespecified baseline variables considered clinically relevant or potentially associated with outcomes: age, sex, Society of Thoracic Surgeons Predicted Risk of Mortality (STS-PROM) score (ranging from 0% to 100%, with higher percentages indicating greater risk of death within 30 days after the surgical procedure), diabetes, prior myocardial infarction, prior PCI, prior coronary artery bypass graft surgery, prior cerebrovascular events, chronic obstructive pulmonary disease, atrial fibrillation, extracardiac arteriopathy, prior overt bleeding requiring medical intervention, mean aortic valve gradient, discharge medications (dual antiplatelet therapy, statin, and β-blocker), and valve type implanted (ACURATE Neo vs SAPIEN 3). When discharge medication was not available (eg, because of death or withdrawal), preadmission medication status was used instead. Missing values were rare; a single missing value for mean gradient was imputed using the cohort mean. The proportional hazards assumption of Cox regression models was evaluated using Schoenfeld residuals and found to be appropriate. All statistical analyses were 2-sided and performed using Stata 18 (StataCorp LLC), with *P* < .05 considered statistically significant. Data were analyzed for the as treated population from February 17 through August 13, 2025.

## Results

### Study Population

Among 739 patients enrolled in SCOPE I between February 8, 2017, and February 2, 2019, 732 who underwent TAVR (ACURATE Neo, n = 364; SAPIEN 3, n = 368) were included in the present post hoc analysis (mean [SD] age, 82 [4] years; 416 [56.8%] female, 316 [43.2%] male; mean [SD] STS-PROM score, 4.3% [2.9%]) (Table 1). Obstructive CAD was observed in 373 patients (51.0%): single-vessel disease in 134 patients (39.9%), 2-vessel disease in 104 patients (31.0%), 3-vessel disease in 98 patients (29.2%), and left main disease in 15 patients (4.5%). Patients with obstructive CAD were less likely to be female (170 [45.6%] vs 246 [68.5%]) and have a higher mean (SD) STS-PROM score (4.5% [3.2%] vs 4.0% [2.4%]), and atherosclerotic comorbidity burden compared with patients without obstructive CAD. Although there were no significant differences in aortic valve area, left ventricular ejection fraction, and the prevalence of significant concomitant valvular disease between groups, patients with obstructive CAD had a lower mean aortic valve gradient at baseline than those without obstructive CAD. There were no differences in the distribution of the type or size of transcatheter heart valves or the frequency of procedural complications (eTable 1 in Supplement 1). Oral anticoagulant and statins were more frequently prescribed at discharge in patients with vs without obstructive CAD.

**Table 1.** Baseline and Procedural Characteristics According to the Presence or Absence of Obstructive CAD

Characteristic	Patients overall					Patients with obstructive CAD			
	All patients (n = 732)	With obstructive CAD (n = 373)	No obstructive CAD (n = 359)	Difference (95% CI), %^a^	*P* value^b^	With PCI (n = 144)	No PCI (n = 229)	Difference (95% CI), %^a^	*P* value^b^
Age, mean (SD) y	82 (4)	82 (4)	82 (4)	0.38 (−0.21 to 0.98)	.20	82 (4)	83 (4)	−0.07 (−0.94 to 0.80)	.87
Sex, No. (%)									
Female	416 (56.8)	170 (45.6)	246 (68.5)	−23 (−30 to −16)		67 (46.5)	103 (45.0)	2 (−9 to 12)	.77
Male	316 (43.2)	203 (54.1)	113 (31.5%)	23 (16 to 30)	<.001	77 (53.5)	126 (55.0)	−2 (−12 to 9)	
Body mass index, mean (SD)^c^	27.6 (4.6)	27.4 (4.6)	27.8 (4.6)	−0.37 (−1.03 to 0.29)	.27	27.5(4.1)	27.4 (4.8)	0.10 (−0.85 to 1.05)	.83
STS-PROM score, mean (SD), %^d^	4.3 (2.9)	4.5 (3.2)	4.0 (2.4)	0.55 (0.13 to 0.96)	.01	4.5 (3.9)	4.6 (2.7)	−0.03 (−0.70 to 0.64)	.94
NYHA class III or IV, No. (%)	549 (75.0)	269 (72.1)	280 (78.0)	−6 (−12 to 0)	.07	101 (70.1)	168 (73.4)	−3 (−13 to 6)	.55
CCS grade III or IV, No. (%)	44 (6.0)	28 (7.5)	16 (4.5)	3 (−0 to 6)	.09	14 (9.7)	14 (6.1)	4 (−2 to 9)	.23
Syncope, No. (%)	81 (11.1)	40 (10.7)	41 (11.4)	−1 (−5 to 4)	.81	12 (8.3)	28 (12.2)	−4 (−10 to 3)	.30
Comorbidity									
Hypertension, No. (%)	667 (91.1)	343 (92.0)	324 (90.3)	2 (−2 to 6)	.44	130 (90.3)	213 (93.0)	−3 (−8 to 3)	.43
Diabetes, No. (%)	219 (29.9)	135 (36.2)	84 (23.4)	13 (6 to 19)	<.001	51 (35.4)	84 (36.7)	−1 (−11 to 9)	.83
Serum creatinine >2 mg/dL, No. (%)	28 (3.8)	16 (4.3)	12 (3.3)	1 (−2 to 4)	.57	5 (3.5)	11 (4.8)	−1 (−6 to 3)	.61
Previous myocardial infarction, No. (%)	84 (11.5)	75 (20.1)	9 (2.5)	18 (13 to 22)	<.001	35 (24.3)	40 (17.5)	7 (−2 to 15)	.11
History of PCI, No. (%)	238 (32.5)	218 (58.4)	20 (5.6)	53 (47 to 58)	<.001	139 (96.5)	79 (34.5)	62 (54 to 70)	<.001
History of CABG, No. (%)	59 (8.1)	49 (13.1)	10 (2.8)	10 (6 to 14)	<.001	11 (7.6)	38 (16.6)	−9 (−16 to −2)	.01
Previous stroke or TIA, No. (%)	92 (12.6)	57 (15.3)	35 (9.7)	6 (1 to 10)	.03	22 (15.3)	35 (15.3)	−0 (−8 to 8)	>.99
COPD, No. (%)	77 (10.5)	30 (8.0)	47 (13.1)	−5 (−9 to −1)	.03	13 (9.0)	17 (7.4)	2 (−4 to 7)	.57
Atrial fibrillation, No. (%)	268 (36.6)	145 (38.9)	123 (34.3)	5 (−2 to 12)	.22	54 (37.5)	91 (39.7)	−2 (−12 to 8)	.74
Extracardiac arteriopathy, No. (%)	84 (11.5)	53 (14.2)	31 (8.6)	6 (1 to 10)	.02	24 (16.7)	29 (12.7)	4 (−3 to 11)	.29
Frailty, No. (%)	120 (16.4)	59 (15.8)	61 (17.0)	−1 (−7 to 4)	.69	33 (22.9)	26 (11.4)	12 (4 to 19)	.004
Previous overt bleeding requiring intervention, No. (%)	26 (3.6)	20 (5.4)	6 (2)	4 (1 to 6)	.008	9 (6.3)	11 (4.8)	1 (−3 to 6)	.64
Hemoglobin nadir post procedure, mean (SD), g/L	102.4 (15.9)	101.8 (16.4)	103.0 (15.)2	−1.22 (−3.53 to 1.08)	.30	98.8 (16.1)	103.7 (16.4)	−4.84 (−8.25 to −1.44)	.005
Anemia post procedure, No. (%)	689 (94.4)	356 (95.7)	333 (93.0)	3 (−1 to 6)	.15	140 (97.2)	216 (94.7)	2 (−2 to 7)	.30
Echocardiography^e^									
Aortic valve area, mean (SD), cm^2^	0.73 (0.19)	0.74 (0.19)	0.72 (0.19)	0.02 (−0.01 to 0.05)	.16	0.74 (0.19)	0.73 (0.19)	0.01 (−0.03 to 0.05)	.79
Aortic valve gradient, mean (SD), mmHg	42.3 (16.3)	40.0 (15.1)	44.6 (17.1)	−4.52 (−6.86 to −2.19)	<.001	40.7 (14.6)	39.6 (15.4)	1.07 (−2.08 to 4.22)	.51
Left ventricular ejection fraction, mean (SD), %	56.9 (10.8)	56.2 (11.0)	57.5 (10.6)	−1.27 (−2.84 to 0.29)	.11	56.2 (11.7)	56.2 (10.5)	0 (−2.30 to 2.29)	>.99
Moderate or severe aortic regurgitation, No. (%)	77 (11.3)	33 (9.7)	44 (13.0)	−3 (−8 to 1)	.18	11 (8.3)	22 (10.5)	−2 (−9 to 4)	.58
Moderate or severe mitral regurgitation, No. (%)	110 (15.1)	59 (15.9)	51 (14.3)	2 (−4 to 7)	.61	22 (15.3)	37 (16.2)	−1 (−9 to 7)	.89
Moderate or severe tricuspid regurgitation, No. (%)	82 (11.6)	38 (10.6)	44 (12.6)	−2 (−7 to 3)	.41	14 (10.1)	24 (10.9)	−1 (−7 to 6)	.86
CAD characteristic, No. (%)									
Single-vessel disease	134 (39.9)	134 (39.9)	NA	NA	NA	42 (38.9)	92 (40.4)	−1 (−13 to 10)	.81
2-Vessel disease	104 (31.0)	104 (31.0)	NA	NA	NA	33 (30.6)	71 (31.1)	−1 (−11 to 10)	>.99
3-Vessel disease	98 (29.2)	98 (29.2)	NA	NA	NA	33 (30.6)	65 (28.5)	2 (−8 to 13)	.70
Left main disease	15 (4.5)	15 (4.5)	NA	NA	NA	4 (3.7)	11 (4.8)	−1 (−6 to 4)	.78
Medication at discharge, No. (%)									
Single antiplatelet therapy	234 (32)	125 (34)	109 (31)	3 (−4 to 10)	.38	44 (31)	81 (36)	−5 (−15 to 5)	.37
Dual antiplatelet therapy	407 (56)	206 (56)	201 (56)	−1 (−8 to 6)	.82	95 (66)	111 (49)	18 (7 to 28)	.001
Oral anticoagulant	291 (40)	163 (44)	128 (36)	8 (1 to 15)	.03	60 (42)	103 (45)	−3 (−14 to 7)	.59
Statin	490 (67)	291 (78)	199 (56)	23 (16 to 29)	<.001	109 (76)	182 (80)	−4 (−12 to 5)	.44
Renin-angiotensin system inhibitor	490 (67)	260 (70)	230 (65)	5 (−1 to 12)	.13	104 (73)	156 (68)	4 (−5 to 14)	.42
β-blocker	476 (65)	254 (68)	222 (62)	6 (−1 to 13)	.09	98 (69)	156 (68)	0 (0 to 0)	>.99
Calcium channel antagonist	253 (35)	133 (36)	120 (34)	2 (−5 to 9)	.59	58 (41)	75 (33)	8 (−2 to 18)	.15
Proton pump inhibitor)	379 (52)	175 (47)	204 (57)	−10 (−17 to −3)	.007	79 (55)	96 (4%)	13 (3 to 24)	.01

Abbreviations: CABG, coronary artery bypass grafting; CAD, coronary artery disease; CCS, Canadian Cardiologists Society; COPD, chronic obstructive pulmonary disease; NA, not applicable; NYHA, New York Heart Association; PCI, percutaneous coronary intervention; STS-PROM, Society of Thoracic Surgeons Predicted Risk of Mortality; TIA, transient ischemic attack.

SI conversions: To convert creatinine to micromoles per liter, multiply by 88.4; hemoglobin to grams per liter, by 10.0.

^a^ Indicates between-group difference (mean difference for continuous variables and proportion difference for categorical variables).

^b^ Counts (%), pairwise *P* values from Fisher exact test; means with SDs, pairwise *P* values from analysis of variance *F* test.

^c^ Body mass index calculated as weight in kilograms divided by height in meters squared.

^d^ STS-PROM scores range from 0% to 100%, with higher percentages indicating greater risk of death within 30 days after the surgical procedure.

^e^ Transthoracic echocardiography (or transesophageal imaging if transthoracic data unavailable); cardiac catheterization data used if no echocardiogram available.

Of 373 patients with obstructive CAD, 144 (38.6%) underwent periprocedural PCI, the majority (137 [95.1%]) prior to TAVR, with a median (IQR) of 41 (17-81) days prior to TAVR. There were no significant differences in patient demographics, atherosclerotic comorbidity burden, distribution of CAD, TAVR procedural characteristics and results between groups, although patients who underwent periprocedural PCI were more likely to be prescribed dual antiplatelet therapy and proton pump inhibitors at discharge.

### Patient-Reported Health Status

**Figure 1.**
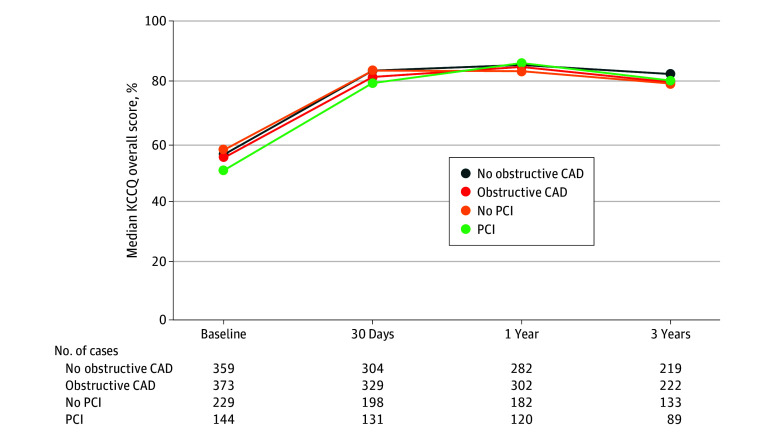
Overall Kansas City Cardiomyopathy Questionnaire (KCCQ) Score Over Time Data in the figure based on descriptive analyses. KCCQ scores range from 0 to 100 with higher numbers indicating better health status. CAD indicates coronary artery disease; PCI, percutaneous coronary intervention.

**Figure 2.**
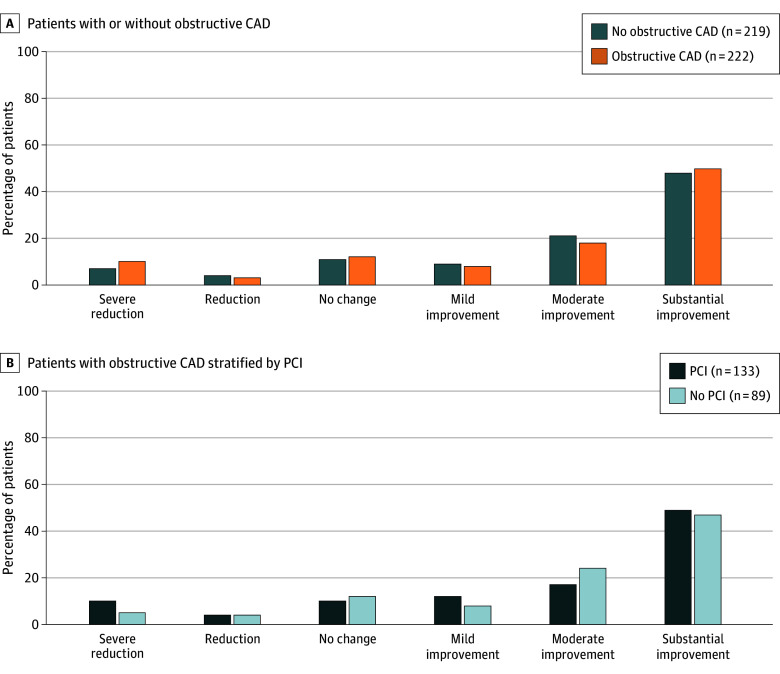
Ordinal Analysis of Overall Kansas City Cardiomyopathy Questionnaire (KCCQ) Score Data in the figure based on descriptive analyses. KCCQ scores range from 0 to 100 with higher numbers indicating better health status. Ordinal analysis of overall KCCQ score was applied based on changes from baseline:^14,21^ severe reduction (by >10 points), reduction (by 5-10 points), no change (change between −5 and 5 points), mild improvement (increase by 5-10 points), moderate improvement (increase by 10-20 points), and substantial improvement (increase ≥20 points). CAD indicates coronary artery disease; PCI, percutaneous coronary intervention.

eTable 2 in Supplement 1 summarizes the KCCQ overall score and its constituent domains throughout the study period. Patients with or without obstructive CAD had similar KCCQ scores throughout the study period (median [IQR] overall KCCQ score at baseline: 54.2 [40.3-69.8] vs 55.2 [38.5-72.9]; at 30 days: 81.2 [64.6-91.7] vs 83.3 [69.4-91.7]; at 1 year: 84.9 [65.6-93.8] vs 85.4 [70.8-92.8]; and at 3 years: 79.7 [64.4-90.6] vs 82.3 [68.2-91.7], respectively) (Figure 1). The change in overall KCCQ score from baseline was similar after TAVR in patients with or without obstructive CAD, accompanied by a similar distribution in ordinal analyses. These results remained consistent when patients with obstructive CAD were stratified according to the use of PCI or conservative management (Figure 2; eTable 3 Supplement 1).

### Clinical Outcomes

Table 2 and eTable 4 in Supplement 1 summarize clinical outcomes according to the presence or absence of obstructive CAD and the use of PCI or conservative management. At the 3-year follow-up, 10 patients (1.4%) were unavailable for follow-up. There was no statistically significant difference in the rates of all-cause or cardiovascular mortality between patients with vs without obstructive CAD at baseline (all-cause death: 88 of 373 [24.7%] vs 76 of 359 [22.3%], adjusted HR, 0.97 [95% CI, 0.66-1.43]; cardiovascular death: 59 of 373 [17.6%] vs 51 of 359 [15.5%], adjusted HR, 0.87 [95% CI, 0.54-1.42]). During the study period, 21 patients experienced myocardial infarction, and 9 patients underwent unplanned PCI, the majority of which occurred beyond the periprocedural period. The presence of obstructive CAD at baseline was associated with a numerically, although not statistically significant, higher risk of myocardial infarction (18 of 372 [5.5%] vs 3 of 359 [1.1%]; adjusted HR, 3.83 [95% CI, 0.96-15.31]) and unplanned PCI (8 of 366 [2.2%] vs 1 of 352 [0.3%]; RR, 7.69 [95% CI, 0.97-61.2]) compared with patients without CAD (Table 2; eFigure 1 in Supplement 1). Similarly, there was no statistically significant difference in clinical outcomes between patients with obstructive CAD who were treated with PCI or conservative management, although PCI was associated a higher, albeit not statistically significant, risk of Bleeding Academic Research Consortium (BARC) type 3 or 5 bleeding (adjusted HR, 1.70 [95% CI, 0.98-2.94]), the majority of which (123 of 169 events, 72.8%) occurred within the periprocedural period (eFigure 2 in Supplement 1).

**Table 2.** Outcomes According to the Presence or Absence of Obstructive CAD

Outcome	Patients overall						Patients with obstructive CAD					
	Obstructive CAD, No. /total No. (%) (n = 373)	No obstructive CAD, No. /total No. (%) (n = 359)	HR or RR (95% CI)^a^	*P* value	Adjusted HR or RR (95% CI)^a^	Adjusted *P* value	PCI, No. /total No. (%) (n = 144)	No PCI, No. /total No. (%) (n = 229)	HR or RR (95% CI)^b^	*P* value	Adjusted HR or RR (95% CI)^b^	Adjusted *P* value
**1-Year outcomes**												
Clinical efficacy	236/334 (70.7)	225/313 (71.9)	0.98 (0.89-1.08)	.73	1.06 (0.94-1.19)	.38	94/128 (73.4)	142/206 (68.9)	1.07 (0.93-1.23)	.38	1.09 (0.90-1.32)	.37
Favorable outcome	240/330 (72.7)	225/298 (75.5)	0.96 (0.88-1.05)	.43	1.03 (0.92-1.15)	.61	93/125 (74.4)	147/205 (71.7)	1.04 (0.91-1.19)	.59	1.09 (0.91-1.30)	.37
Acceptable outcome	23/330 (7.0)	19/298 (6.4)	1.09 (0.61-1.96)	.77	0.79 (0.39-1.58)	.50	13/125 (10.4)	10/205 (4.9)	2.13 (0.96-4.71)	.06	2.60 (0.81-8.37)	.11
Unfavorable outcome	67/330 (20.3)	54/298 (18.1)	1.12 (0.81-1.55)	.49	0.98 (0.68-1.43)	.93	19/125 (15.2)	48/205 (23.4)	0.65 (0.40-1.05)	.07	0.60 (0.33-1.07)	.08
All-cause mortality	37/373 (10.1)	28/359 (7.9)	1.26 (0.77-2.06)	.35	1.19 (0.65-2.17)	.57	10/144 (7.1)	27/229 (11.9)	0.58 (0.28-1.19)	.14	0.70 (0.28-1.74)	.44
Cardiovascular mortality	20/373 (5.5)	19/359 (5.5)	1.01 (0.54-1.89)	.98	0.85 (0.38-1.88)	.69	8/144 (5.7)	12/229 (5.4)	1.04 (0.42-2.54)	.93	1.13 (0.34-3.76)	.84
All stroke	15/372 (4.1)	17/359 (4.9)	0.85 (0.42-1.70)	.64	0.87 (0.36-2.12)	.76	4/144 (2.8)	11/228 (4.9)	0.57 (0.18-1.80)	.34	0.36 (0.09-1.40)	.14
Hospitalization for valve-related dysfunction or heart failure	30/372 (8.5)	39/359 (11.4)	0.72 (0.45-1.16)	.18	0.65 (0.36-1.16)	.15	13/144 (9.6)	17/228 (7.9)	1.21 (0.59-2.50)	.60	2.07 (0.70-6.11)	.19
Myocardial infarction	12/372 (3.4)	0	NA	NA	NA	NA	4/144 (2.8)	8/228 (3.8)	0.78 (0.23-2.59)	.68	0.60 (0.13-2.67)	.50
All bleeding	113/372 (30.6)	90/359 (25.3)	1.22 (0.93-1.62)	.15	1.07 (0.75-1.51)	.71	51/144 (35.7)	62/228 (27.4)	1.39 (0.96-2.01)	.08	1.60 (0.97-2.66)	.07
BARC type 2 bleeding	34/372 (10.2)	32/359 (9.7)	1.04 (0.64-1.69)	.86	0.73 (0.40-1.36)	.33	14/144 (10.8)	20/228 (9.8)	1.17 (0.59-2.31)	.66	1.20 (0.48-2.99)	.69
BARC type 3 or 5 bleeding	84/372 (23.8)	62/359 (18.2)	1.33 (0.95-1.84)	.09	1.23 (0.82-1.86)	.32	39/144 (28.8)	45/228 (20.6)	1.46 (0.95-2.23)	.09	1.72 (0.96-3.10)	.07
**3-Year outcomes**												
Clinical efficacy	163/313 (52.1)	159/298 (53.4)	0.98 (0.84-1.14)	.75	1.10 (0.92-1.32)	.29	65/125 (52.0)	98/188 (52.1)	1.00 (0.80-1.24)	.98	0.97 (0.73-1.30)	.86
Favorable outcome	170/302 (56.3)	171/284 (60.2)	0.93 (0.81-1.07)	.34	1.02 (0.87-1.20)	.81	63/120 (52.5)	107/182 (58.8)	0.89 (0.72-1.10)	.28	0.90 (0.69-1.17)	.44
Acceptable outcome	21/302 (7.0)	11/284 (3.9)	1.80 (0.88-3.67)	.10	1.59 (0.65-3.84)	.31	12/120 (10.0)	9/182 (4.9)	2.02 (0.88-4.65)	.09	2.91 (1.15-7.35)	.02
Unfavorable outcome	111/302 (36.8)	102/284 (35.9)	1.02 (0.82-1.26)	.83	0.95 (0.74-1.21)	.66	45/120 (37.5)	66/182 (36.3)	1.03 (0.76-1.39)	.83	1.02 (0.68-1.52)	.94
All-cause mortality	88/373 (24.7)	76/359 (22.3)	1.13 (0.83-1.53)	.45	0.97 (0.66-1.43)	.88	36/144 (26.3)	52/229 (23.7)	1.09 (0.71-1.66)	.70	1.07 (0.60-1.90)	.82
Cardiovascular mortality	59/373 (17.6)	51/359 (15.5)	1.13 (0.78-1.64)	.53	0.87 (0.54-1.42)	.58	27/144 (20.5)	32/229 (15.6)	1.32 (0.79-2.21)	.28	1.31 (0.65-2.65)	.45
All stroke	20/372 (5.5)	21/359 (6.0)	0.92 (0.50-1.69)	.78	1.25 (0.58-2.67)	.57	5/144 (3.5)	15/228 (6.7)	0.53 (0.19-1.45)	.21	0.47 (0.14-1.61)	.23
Hospitalization for valve-related dysfunction or heart failure	53/372 (16.3)	57/359 (17.4)	0.89 (0.61-1.29)	.54	0.75 (0.47-1.20)	.23	22/144 (17.3)	31/228 (15.6)	1.14 (0.66-1.97)	.64	1.82 (0.85-3.91)	.12
Myocardial infarction	18/372 (5.5)	3/359 (1.1)	5.98 (1.76-20.30)	.004	3.83 (0.96-15.31)	.06	7/144 (5.6)	11/228 (5.5)	0.98 (0.38-2.53)	.97	0.86 (0.24-3.05)	.81
Unplanned PCI	8/366 (2.2)	1/352 (0.3)	7.69 (0.97-61.20)	.02	NA	NA	1/141 (0.7)	7/225 (3.1)	0.23 (0.03-1.83)	.13	NA	NA
All bleeding	130/372 (36.3)	102/359 (29.2)	1.26 (0.97-1.63)	.08	1.11 (0.80-1.53)	.55	56/144 (40.2)	74/228 (33.8)	1.29 (0.91-1.82)	.15	1.38 (0.87-2.19)	.17
BARC type 2 bleeding	41/372 (13.2)	38/359 (12.1)	1.08 (0.69-1.67)	.75	0.78 (0.45-1.37)	.39	16/144 (13.1)	25/228 (13.1)	1.08 (0.58-2.03)	.81	1.01 (0.45-2.24)	.98
BARC type 3 or 5 bleeding	97/372 (28.6)	72/359 (21.9)	1.33 (0.98-1.81)	.06	1.26 (0.86-1.85)	.24	45/144 (34.7)	52/228 (24.9)	1.46 (0.98-2.17)	.06	1.70 (0.98-2.94)	.06

Abbreviations: BARC, Bleeding Academic Research Consortium; CAD, coronary artery disease; HR, hazard ratio; NA, not applicable; PCI, percutaneous coronary intervention; RR, risk ratio.

^a^ Obstructive CAD vs no obstructive CAD.

^b^ PCI vs no PCI.

### Integrated Clinical Outcomes and Patient-Reported Health Status

**Figure 3.**
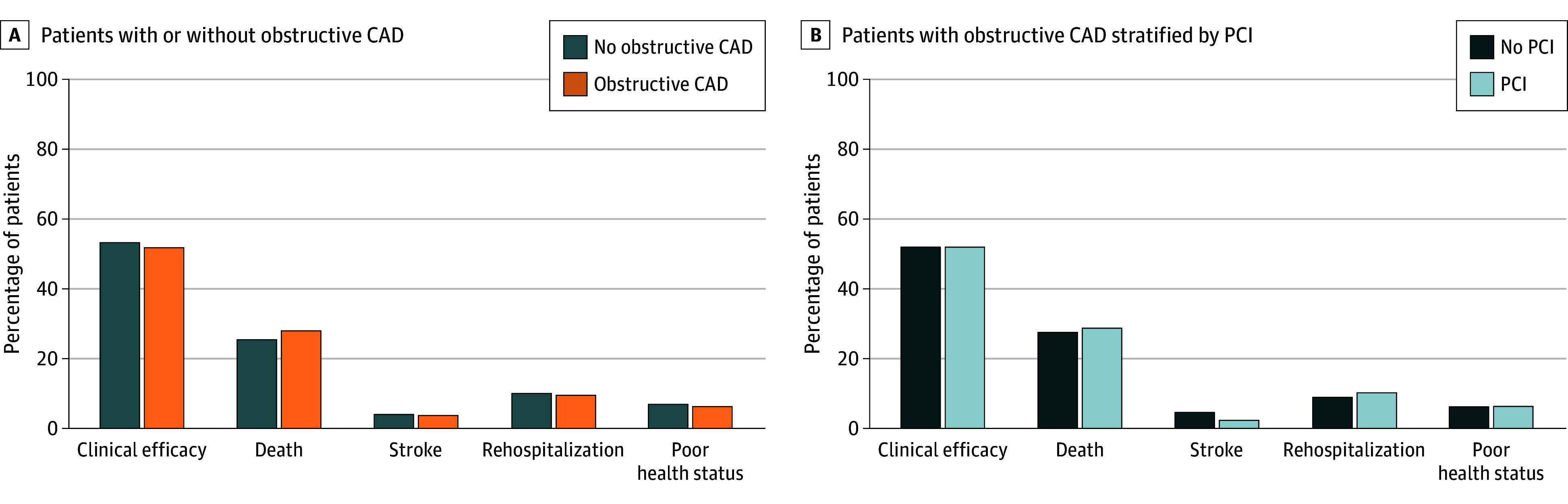
Clinical Efficacy of Transcatheter Aortic Valve Replacement at 3 Years Bar graphs show the percentages of patients who achieved clinical efficacy of transcatheter aortic valve replacement and, among those who did not, the hierarchical distribution of associated adverse events, including death, stroke, rehospitalization for valve-related dysfunction or heart failure, and poor health status (Kansas City Cardiomyopathy Questionnaire [KCCQ] overall score <45 or decline >10 points from baseline). KCCQ scores range from 0 to 100 with higher numbers indicating better health status. CAD indicates coronary artery disease; PCI, percutaneous coronary intervention.

Integrated clinical outcomes and patient-reported health status end points were assessed in 334 of 373 (89.5%) patients and 313 of 373 (83.9%) patients with obstructive CAD, and in 313 of 359 (87.2%) patients and 298 of 359 (83.0%) of patients without obstructive CAD at 1 and 3 years, respectively (Table 2). Overall clinical efficacy was achieved in 236 of 334 (70.7%) of patients with obstructive CAD vs 225 of 313 (71.9%) of patients without obstructive CAD at 1 year, and in 163 of 313 (52.1%) of patients with obstructive CAD vs 159 of 298 (53.4%) of patients without obstructive CAD at 3 years. The presence of obstructive CAD was not associated with reduced clinical efficacy throughout the study period (1 year: adjusted RR, 1.06 [95% CI, 0.94-1.19]; 3 years: adjusted RR, 1.10 [95% CI, 0.92-1.32]) (Figure 3). Furthermore, there was no difference in integrated clinical outcomes and patient-reported health status end points between patients with obstructive CAD who were treated with PCI or conservative management.

## Discussion

The salient findings of this post hoc analysis of the SCOPE I randomized clinical trial can be summarized as follows. Obstructive CAD (defined as at least 1 major epicardial coronary vessel with >50% diameter stenosis) was present in about half of patients with severe AS undergoing TAVR. Patient-reported health status (assessed by overall KCCQ score) was comparable in patients with or without obstructive CAD throughout the study period, with similar improvements following TAVR. Obstructive CAD showed a higher frequency of unplanned PCI and myocardial infarction after TAVR; however, no clear association with mortality or reduced clinical efficacy of TAVR was observed. In addition, periprocedural PCI among patients with obstructive CAD was not associated with improved clinical outcomes, patient-reported health status, or integrated clinical outcomes and quality of life end points.

Although the reported prevalence of obstructive CAD among patients undergoing TAVR varies widely (largely related to varying definitions), patients with severe AS and obstructive CAD have a higher risk profile due to the presence of cardiovascular risk factors that can complicate disease management. Recent studies have reported that untreated obstructive CAD is associated with an increased risk of coronary events, although its association with long-term prognosis following TAVR remains undetermined. A single-center observational study reported that although patients with obstructive CAD had higher rates of acute coronary syndrome and unplanned coronary revascularization, there was no association with major adverse cardiovascular events or all-cause mortality, irrespective of the severity or extent of CAD.^6^ In the Nordic Aortic Valve Intervention (NOTION)-3 trial,^9^ TAVR plus complete percutaneous revascularization had a lower incidence of the primary composite end point of death from any cause, myocardial infarction, or urgent revascularization than TAVR with conservative management. Of note, this difference was driven by lower rates of myocardial infarction or urgent revascularization, with no difference observed in all-cause mortality. Furthermore, the association of PCI with the primary end point was pronounced in patients with 90% or more diameter stenosis but not in patients with less severe disease.^9^ Consistent with these previous studies, our analysis showed that patients with obstructive CAD experienced more frequent myocardial infarction and unplanned PCI, whereas mortality was comparable with patients without obstructive CAD. However, periprocedural PCI was not clearly associated with a reduction in ischemic events throughout the follow-up period in our study. Importantly, routine PCI is also associated with an increased risk of bleeding in patients undergoing TAVR.^9,22^ Indeed, three-quarters of BARC type 3 or 4 bleeding events in this study occurred during the periprocedural period. These findings suggest that the clinical benefit of PCI is not universal in this cohort.

In the present analysis, we observed a similar overall KCCQ score (and extent of score improvement) among patients with or without obstructive CAD at all time points, regardless of the use of periprocedural PCI. A previous study reported that angina has low sensitivity, specificity, and positive and negative predictive values for the identification of CAD (defined as 70% or more luminal diameter narrowing of an epicardial coronary artery) in the setting of severe AS.^23^ Although symptomatic status is a key factor in treatment decisions, distinction between CAD and AS as the source of symptoms is challenging due to overlapping features (such as exertional dyspnea) and limitations in exercise tolerance. These diagnostic challenges complicate clinical decision-making and hinder determination of the optimal management strategy in this population.

Health status assessments may be biased over time, since patients who derive least benefit from treatment are also more likely to die. In the present study, we applied VARC-3 integrated end points to comprehensively evaluate treatment benefit at the individual patient level.^14,24,25^ In our descriptive analysis, patients with or without obstructive CAD had comparable rates of favorable outcome and clinical efficacy during the 3 years following TAVR, regardless of the need for periprocedural PCI. These findings suggest that the presence of obstructive CAD does not in principle compromise the overall benefits of TAVR. Historically, obstructive CAD was believed to be associated with increased risk of ischemic events during and after TAVR, while coronary revascularization was assumed to improve procedural safety and prognosis.^26^ However, emerging evidence highlights the need for an individualized approach that accounts for symptoms, quality of life, life expectancy, and overall health status.^1^ For many patients with severe AS and obstructive CAD, isolated TAVR with optimal medical therapy may be a reasonable strategy—particularly for patients without high-grade proximal coronary stenosis (>90% stenosis), asymptomatic or oligosymptomatic patients after TAVR, and patients with high morbidity or elevated bleeding risk. Our study supports further refinement of management strategies in this population that incorporate both clinical outcomes and patient-reported health status. Several ongoing clinical trials aim to determine the optimal treatment approach in patients with combined AS and CAD (ie, A Randomized, Comparative Effectiveness Study of Staged Complete Revascularization With Percutaneous Coronary Intervention to Treat Coronary Artery Disease vs Medical Management Alone in Patients With Symptomatic Aortic Valve Stenosis Undergoing Elective Transfemoral Transcatheter Aortic Valve Replacement: The COMPLETE TAVR Study^27^ Functional Assessment in TAVI: FAITAVI^28^ Optimal Timing of Transcatheter Aortic Valve Implantation and Percutaneous Coronary Intervention - The TAVI PCI Trial^29^ Percutaneous Coronary Intervention Before Transcatheter Aortic Valve Implantation: the PRO-TAVI Trial^30^ and Randomized Trial of Coronary Artery Disease Assessment Strategies in Patients With Severe Aortic Stenosis Undergoing Transcatheter Aortic Valve Implantation^31^). Their outcomes will be instrumental in shaping future guidelines for the treatment of patients with severe AS and obstructive CAD.

### Limitations

The results of the present study should be interpreted in the light of several limitations. First, this was a post hoc observational analysis of the SCOPE I trial and, therefore, hypothesis-generating by design; the findings should be interpreted descriptively and cannot determine the relationship between obstructive CAD and clinical outcomes after TAVR. Second, although we provide comprehensive data from an established randomized clinical trial with high data quality standards and independent event adjudication, KCCQ scores were unavailable for a modest proportion of patients. While the proportion of missing data in the present analysis is consistent with the intermediate-risk trials,^32,33^ this underreporting of KCCQ scores may have introduced responder bias, as sicker patients are less likely to complete questionnaires.^25^ Purposeful approaches to minimize missing data from patient-reported outcome instruments should be considered.^34,35^ Third, although the severity of obstructive CAD was evaluated within standard periprocedural assessment protocols, this assessment was undertaken independently by Heart Teams at individual participating centers without core laboratory analysis. Furthermore, more granular definitions of disease severity, extent, and complexity that were not prespecified in SCOPE I could not be included. For the same reason, we were unable to evaluate PCI strategies, (eg, physiology-guided PCI or complete revascularization). The decision to perform PCI was entirely at the discretion of the treating physician and may have been influenced by the CAD characteristics. However, the clinical impact of these CAD characteristics and revascularization strategies on this population are still under investigation, and the findings reflect the outcomes as achieved in clinical practice. Fourth, the follow-up period in SCOPE I was limited to 3 years after TAVR, which may not fully capture the long-term impact of coronary revascularization on cardiovascular mortality.^36,37^ Finally, the ACURATE platform has recently been discontinued, which may diminish the direct applicability of our findings to contemporary and future clinical practice.

## Conclusions

In this post hoc analysis of the SCOPE I randomized clinical trial, approximately half of the patients with severe AS undergoing TAVR had obstructive CAD, and these patients demonstrated similar status and improvement in overall KCCQ score after TAVR to patients without obstructive CAD. Whereas obstructive CAD showed a higher frequency of coronary events after TAVR, there was no clear association with mortality or reduced clinical efficacy of TAVR. Furthermore, periprocedural PCI in patients with obstructive CAD did not improve clinical outcomes, patient-reported health status, or integrated clinical outcomes and health status end points. These findings suggest that a nuanced, tailored approach is essential in the treatment of TAVR candidates with the commonly encountered combination of severe AS and CAD.

## Notes

Supplement 1. **eTable 1.** Procedural characteristics and complications **eTable 2.** KCCQ score over time **eTable 3.** Changes in health status compared to overall KCCQ score at baseline **eTable 4.** Clinical outcomes at 30 days after TAVR **eFigure 1.** Cumulative incidence of clinical outcomes according to the presence or absence of obstructive CAD **eFigure 2.** Cumulative incidence of clinical outcomes in patients with obstructive CAD according to use of PCI

Supplement 2.
Data Sharing Statement
