# Supplementary material for: Obstructive Coronary Artery Disease and Health Status in Transcatheter Aortic Valve Replacement: A Post Hoc Analysis of the SCOPE I Randomized Clinical Trial
Source: JAMA Netw Open. 2025 Dec 9;8(12):e2547111. doi: 10.1001/jamanetworkopen.2025.47111 (PMC12690430; doi:10.1001/jamanetworkopen.2025.47111)
Supplement: Supplement 1. — eTable 1. Procedural characteristics and complications eTable 2. KCCQ score over time eTable 3. Changes in health status compared to overall KCCQ score at baseline eTable 4. Clinical outcomes at 30 days after TAVR eFigure 1. Cumulative incidence of clinical outcomes according to the presence or absence of obstructive CAD eFigure 2. Cumulative incidence of clinical outcomes in patients with obstructive CAD according to use of PCI [file jamanetwopen-e2547111-s001.pdf]

Supplementary Online Content

Tomii D, Lanz J, Thiele H, et al. Obstructive coronary artery disease and health status in transcatheter aortic valve replacement: a post hoc analysis of the Scope I randomized clinical trial. *JAMA Netw Open*. 2025;8(12):e2547111. doi:10.1001/jamanetworkopen.2025.47111

eTable 1. Procedural characteristics and complications
eTable 2. KCCQ score over time
eTable 3. Changes in health status compared to overall KCCQ score at baseline
eTable 4. Clinical outcomes at 30 days after TAVR
eFigure 1. Cumulative incidence of clinical outcomes according to the presence or absence of obstructive CAD
eFigure 2. Cumulative incidence of clinical outcomes in patients with obstructive CAD according to use of PCI

This supplementary material has been provided by the authors to give readers additional information about their work.

© 2025 Tomii D et al. *JAMA Network Open*.

eTable 1. Procedural characteristics and complications

	Overall					Patients with obstructive CAD			
	**All patients N = 732**	**Obstructive CAD N = 373**	**No obstructive CAD N = 359**	**Difference (95% CI)**	**P value**	**PCI N = 144**	**No PCI N = 229**	**Difference (95% CI)**	**P value**
**Procedural characteristics**									
General anesthesia, n (%)	160 (21.9%)	58 (15.5%)	102 (28.4%)	-13% (-19% to -7%)	<0.001	27 (18.8%)	31 (13.5%)	5% (-2% to 13%)	0.19
Valve type, n (%)					0.51				0.81
SAPIEN 3	368 (50.3%)	192 (51.5%)	176 (49.0%)	2% (-5% to 10%)	0.55	73 (50.7%)	119 (52.0%)	-1% (-12% to 9%)	0.83

© 2025 Tomii D et al. *JAMA Network Open*.

Acurate Neo	364 (49.7%)	181 (48.5%)	183 (51.0%)	-2% (-10% to 5%)	0.55	71 (49.3%)	110 (48.0%)	1% (-9% to 12%)	0.83
Valve size, mm	25.1 ± 1.6	25.4 ± 1.6	24.9 ± 1.6	0.45 (0.21 to 0.68)	<0.001	25.3 ± 1.6	25.4 ± 1.6	-0.12 (-0.45 to 0.21)	0.48
Moderate or severe aortic regurgitation post-TAVR, n (%)	27 (3.8%)	12 (3.3%)	15 (4.3%)	-1% (-4% to 2%)	0.56	5 (3.6%)	7 (3.1%)	0% (.% to .%)	>0.99
**Procedural complications within 30 days**									
Valve malposition, n (%)	7 (1.0%)	5 (1.3%)	2 (0.6%)	1% (-1% to 2%)	0.45	2 (1.4%)	3 (1.3%)	0% (.% to .%)	>0.99
Coronary artery occlusion requiring intervention, n (%)	1 (0.1%)	0 (0%)	1 (0.3%)	-0% (-1% to 0%)	0.49	0 (0%)	0 (0%)		
Peri-procedural myocardial infarction, n (%)	4 (0.6%)	4 (1.1%)	0 (0%)	1% (-0% to 2%)	0.12	2 (1.4%)	2 (0.9%)	1% (-2% to 3%)	0.64

© 2025 Tomii D et al. *JAMA Network Open*.

Implantation of multiple valves, n (%)	13 (1.8%)	6 (1.6%)	7 (1.9%)	-0% (-2% to 2%)	0.79	3 (2.1%)	3 (1.3%)	1% (-2% to 3%)	0.68
Cardiac tamponade, n (%)	9 (1.2%)	4 (1.1%)	5 (1.4%)	-0% (-2% to 1%)	0.75	0 (0%)	4 (1.8%)	-2% (-4% to 0%)	0.30
Annular rupture, n (%)	1 (0.1%)	0 (0%)	1 (0.3%)	-0% (-1% to 0%)	0.49	0 (0%)	0 (0%)	NA	NA
Left ventricular perforation, n (%)	1 (0.1%)	0 (0%)	1 (0.3%)	-0% (-1% to 0%)	0.49	0 (0%)	0 (0%)	NA	NA
Conversion to open heart surgery, n (%)	3 (0.4%)	1 (0.3%)	2 (0.6%)	-0% (-1% to 1%)	0.62	1 (0.7%)	0 (0%)	1% (-0% to 2%)	0.39
Vascular access complication, n (%)	123 (17.0%)	67 (18.1%)	56 (15.8%)	2% (-3% to 8%)	0.43	30 (21.0%)	37 (16.2%)	5% (-3% to 13%)	0.27
Bleeding, n (%)	175 (24.1%)	97 (26.1%)	78 (22.0%)	4% (-2% to 10%)	0.22	44 (30.8%)	53 (23.2%)	8% (-2% to 17%)	0.12
Immediate procedural death, n (%)	4 (0.6%)	2 (0.5%)	2 (0.6%)	-0% (-1% to 1%)	>0.99	1 (0.7%)	1 (0.4%)	NA	NA

© 2025 Tomii D et al. *JAMA Network Open*.

Moderate or severe paravalvular regurgitation at 30 days, n (%)	44 (6.1%)	17 (4.6%)	27 (7.6%)	-3% (-6% to 1%)	0.12	8 (5.6%)	9 (4.0%)	2% (-3% to 6%)	0.46

Depicted are counts (%, pairwise p-values from Fisher's exact test) or means with standard deviations SD (±SD, pairwise p-values from ANOVA F-test).

Difference (95% CI) indicates between-group difference (mean difference for continuous variables and proportion difference for categorical variables).

Abbreviations as in **Table 1**.

© 2025 Tomii D et al. *JAMA Network Open*.

eTable 2. KCCQ score over time

	Overall					Patients with obstructive CAD			
	All patients N = 732	Obstructive CAD N = 373	No obstructive CAD N = 359	Hodges-Lehmann median difference (95% CI)	P value	PCI N = 144	No PCI N = 229	Hodges-Lehmann median difference (95% CI)	P value
**At baseline**	**N = 692**	**N = 355**	**N = 337**			**N = 135**	**N = 220**		
Overall KCCQ score	54.9 (39.6-71.9)	54.2 (40.3-69.8)	55.2 (38.5-72.9)	1.0% (-2.6%-4.2%)	0.67	50.0 (39.5-66.7)	56.9 (40.9-71.9)	3.1% (-1.5%-8.3%)	0.17
Physical limitation	58.3 (33.3-75.0)	50.0 (33.3-75.0)	58.3 (33.3-75.0)	0.0% (-8.3%-4.2%)	0.75	50.0 (33.3-75.0)	58.0 (37.5-83.3)	0.0% (0.0%-8.3%)	0.25
Quality of life	50.0 (25.0-62.5)	50.0 (25.0-62.5)	50.0 (25.0-62.5)	0.0% (0.0%-0.0%)	0.96	50.0 (25.0-62.5)	50.0 (25.0-62.5)	0.0% (0.0%-12.5%)	0.83
Social limitation	58.3 (33.3-83.3)	50.0 (33.3-75.0)	58.3 (33.3-83.3)	0.0% (0.0%-8.3%)	0.14	50.0 (33.3-75.0)	50.0 (33.3-75.0)	0.0% (-0.0%-8.3%)	0.50

© 2025 Tomii D et al. *JAMA Network Open.*

Symptom frequency	64.6 (46.4-79.2)	66.7 (45.8-79.2)	62.5 (47.9-79.2)	0.0% (-4.2%-2.1%)	0.62	62.5 (39.6-75.0)	66.7 (50.0-79.2)	4.2% (0.0%-10.1%)	0.08
**At 30 days**	**N = 633**	**N = 329**	**N = 304**			**N = 131**	**N = 198**		
Overall KCCQ score	81.8 (67.7-91.7)	81.2 (64.6-91.7)	83.3 (69.4-91.7)	0.0% (-2.1%-3.1%)	0.77	79.2 (61.5-90.6)	83.3 (68.6-91.7)	3.1% (-0.0%-7.3%)	0.08
Physical limitation	83.3 (58.3-100.0)	83.3 (58.3-100.0)	75.0 (58.3-91.7)	0.0% (-8.3%-0.0%)	0.09	83.3 (50.0-97.9)	83.3 (66.7-100.0)	0.0% (0.0%-8.3%)	0.11
Quality of life	87.5 (62.5-100.0)	87.5 (62.5-100.0)	87.5 (75.0-100.0)	0.0% (0.0%-0.0%)	0.26	75.0 (62.5-100.0)	87.5 (62.5-100.0)	0.0% (0.0%-12.5%)	0.24
Social limitation	83.3 (66.7-100.0)	83.3 (62.5-100.0)	87.5 (66.7-100.0)	0.0% (0.0%-4.2%)	0.08	83.3 (58.3-100.0)	83.3 (66.7-100.0)	0.0% (0.0%-8.3%)	0.08
Symptom frequency	83.3 (68.8-95.8)	83.3 (66.7-95.8)	83.3 (70.8-95.8)	0.0% (0.0%-4.1%)	0.57	81.2 (66.7-95.8)	83.3 (68.2-95.8)	0.0% (0.0%-4.2%)	0.33
**At 1 year**	**N = 584**	**N = 302**	**N = 282**			**N = 120**	**N = 182**		

© 2025 Tomii D et al. *JAMA Network Open.*

Overall KCCQ score	85.4 (68.9-93.7)	84.9 (65.6-93.8)	85.4 (70.8-92.8)	0.0% (-2.1%-2.1%)	0.98	85.9 (63.5-93.8)	83.3 (68.8-93.8)	1.0% (-2.6%-4.2%)	0.67
Physical limitation	83.3 (58.3-100.0)	83.3 (58.3-100.0)	75.0 (58.3-91.7)	0.0% (-8.3%-0.0%)	0.10	83.3 (56.2-100.0)	89.6 (62.5-100.0)	0.0% (0.0%-8.3%)	0.51
Quality of life	87.5 (75.0-100.0)	87.5 (75.0-100.0)	87.5 (75.0-100.0)	0.0% (0.0%-0.0%)	0.78	87.5 (62.5-100.0)	87.5 (75.0-100.0)	0.0% (0.0%-0.0%)	0.50
Social limitation	91.7 (66.7-100.0)	91.7 (66.7-100.0)	91.7 (66.7-100.0)	0.0% (0.0%-0.0%)	0.81	91.7 (66.7-100.0)	91.7 (75.0-100.0)	0.0% (0.0%-0.0%)	0.66
Symptom frequency	83.3 (70.8-100.0)	83.3 (70.8-95.8)	85.4 (72.9-100.0)	2.1% (0.0%-4.2%)	0.03	83.3 (70.8-93.8)	83.3 (70.3-100.0)	0.0% (-0.0%-4.2%)	0.46
**At 3 years**	**N = 441**	**N = 222**	**N = 219**			**N = 89**	**N = 133**		
Overall KCCQ score	81.2 (66.7-91.7)	79.7 (64.4-90.6)	82.3 (68.2-91.7)	1.0% (-1.6%-4.2%)	0.43	80.2 (63.8-89.6)	79.2 (64.6-91.7)	0.0% (-4.7%-4.2%)	>0.99
Physical limitation	83.3 (58.3-91.7)	83.3 (58.3-100.0)	75.0 (58.3-91.7)	0.0% (-4.2%-0.0%)	0.78	83.3 (50.0-100.0)	83.3 (58.3-94.0)	0.0% (-8.3%-0.0%)	0.58

© 2025 Tomii D et al. *JAMA Network Open*.

Quality of life	75.0 (62.5-100.0)	75.0 (62.5-87.5)	87.5 (62.5-100.0)	0.0% (0.0%-0.0%)	0.11	75.0 (62.5-100.0)	75.0 (62.5-87.5)	0.0% (0.0%-0.0%)	0.93
Social limitation	83.3 (66.7-100.0)	83.3 (66.7-100.0)	83.3 (66.7-100.0)	0.0% (0.0%-0.0%)	0.91	83.3 (66.7-100.0)	83.3 (66.7-100.0)	0.0% (-0.1%-4.2%)	0.91
Symptom frequency	83.3 (70.8-95.8)	83.3 (66.7-95.8)	87.5 (73.0-95.8)	0.0% (0.0%-4.2%)	0.18	83.3 (70.8-95.8)	83.3 (64.6-95.8)	0.0% (-4.2%-2.1%)	0.44

Values are median (25%-75% interquartile ranges) unless otherwise indicated. This table is based on a descriptive analysis.

KCCQ = Kansas City Cardiomyopathy Questionnaire. Other abbreviations as in **Table 1**.

© 2025 Tomii D et al. *JAMA Network Open*.

eTable 3. Changes in health status compared to overall KCCQ score at baseline

	Overall					Patients with obstructive CAD			
	**All patients N = 732**	**Obstructive CAD N = 373**	**No obstructive CAD N = 359**	**Difference (95% CI)**	**P value**	**PCI N = 144**	**No PCI N = 229**	**Difference (95% CI)**	**P value**
**At 1 year**	**N = 584**	**N = 302**	**N = 282**			**N = 120**	**N = 182**		
Overall KCCQ score ≥60, n (%)	495 (85%)	252 (83%)	243 (86%)	-3% (-9% to 3%)	0.42	99 (82%)	153 (84%)	-2% (-10% to 7%)	0.75
Overall KCCQ score ≥45, n (%)	554 (95%)	281 (93%)	273 (97%)	-4% (-7% to -0%)	0.06	113 (94%)	168 (92%)	2% (-4% to 8%)	0.65
Overall KCCQ score <45, n (%)	30 (5%)	21 (7%)	9 (3%)	4% (0% to 7%)	0.06	7 (6%)	14 (8%)	-2% (-8% to 4%)	0.65
Change in KCCQ Overall Score	23.8 ± 23.2	23.3 ± 22.4	24.5 ± 24.1	-1.23 (-5.10 to 2.63)	0.53	24.9 ± 22.8	22.2 ± 22.1	2.72 (-2.59 to 8.02)	0.32

© 2025 Tomii D et al. *JAMA Network Open.*

Severe reduction, n (%)	36 (6%)	17 (6%)	19 (7%)	-1% (-5% to 3%)	0.61	5 (4%)	12 (7%)	-2% (-8% to 3%)	0.46
Reduction, n (%)	14 (3%)	5 (2%)	9 (3%)	-2% (-4% to 1%)	0.28	2 (2%)	3 (2%)	0% (0% to 0%)	>0.99
No change, n (%)	58 (10%)	31 (11%)	27 (10%)	1% (-4% to 6%)	0.89	11 (10%)	20 (11%)	-2% (-9% to 6%)	0.85
Mild improvement, n (%)	40 (7%)	28 (10%)	12 (4%)	5% (1% to 9%)	0.02	14 (12%)	14 (8%)	4% (-3% to 11%)	0.23
Moderate improvement, n (%)	99 (18%)	50 (17%)	49 (18%)	-1% (-7% to 5%)	0.83	19 (17%)	31 (18%)	-1% (-10% to 8%)	>0.99
Substantial improvement, n (%)	312 (56%)	159 (55%)	153 (57%)	-2% (-10% to 6%)	0.67	62 (55%)	97 (55%)	0% (0% to 0%)	>0.99
**At 3 years**									
Overall KCCQ score ≥60, n (%)	371 (84%)	180 (81%)	191 (87%)	-6% (-13% to 1%)	0.09	69 (78%)	111 (83%)	-6% (-16% to 5%)	0.30

© 2025 Tomii D et al. *JAMA Network Open*.

Overall KCCQ score ≥45, n (%)	413 (94%)	206 (93%)	207 (95%)	-2% (-6% to 3%)	0.56	84 (94%)	122 (92%)	3% (-4% to 10%)	0.60
Overall KCCQ score <45, n (%)	28 (6%)	16 (7%)	12 (5%)	2% (-3% to 6%)	0.56	5 (6%)	11 (8%)	-3% (-10% to 4%)	0.60
Change in KCCQ Overall Score	20.5 ± 22.3	20.2 ± 21.1	20.9 ± 23.6	-0.67 (-4.95 to 3.60)	0.76	21.5 ± 23.4	19.3 ± 19.6	2.13 (-3.69 to 7.95)	0.47
Severe reduction, n (%)	35 (8%)	15 (7%)	20 (10%)	-3% (-8% to 3%)	0.38	8 (10%)	7 (5%)	4% (-3% to 11%)	0.28
Reduction, n (%)	14 (3%)	8 (4%)	6 (3%)	1% (-3% to 4%)	0.79	3 (4%)	5 (4%)	-0% (-6% to 5%)	1.0
No change, n (%)	48 (11%)	24 (11%)	24 (12%)	-0% (-6% to 6%)	1.0	8 (10%)	16 (12%)	-3% (-12% to 6%)	0.66
Mild improvement, n (%)	37 (9%)	20 (9%)	17 (8%)	1% (-4% to 7%)	0.73	10 (12%)	10 (8%)	4% (-4% to 12%)	0.34
Moderate improvement, n (%)	83 (20%)	45 (21%)	38 (18%)	3% (-5% to 10%)	0.54	14 (17%)	31 (24%)	-7% (-18% to 4%)	0.23

© 2025 Tomii D et al. *JAMA Network Open*.

Substantial improvement, n (%)	205 (49%)	102 (48%)	103 (50%)	-2% (-11% to 8%)	0.77	41 (49%)	61 (47%)	2% (-12% to 16%)	0.89

Values are median (25%-75% interquartile ranges) unless otherwise indicated. This table is based on a descriptive analysis.

Difference (95% CI) indicates between-group difference (proportion difference for categorical variables).

Abbreviations as in **Table 1**.

© 2025 Tomii D et al. *JAMA Network Open*.

eTable 4. Clinical outcomes at 30 days after TAVR

	Overall						Patients with obstructive CAD					
	Obstructive CAD N = 373	No obstructive CAD N = 359	Hazard or risk ratios (95% CI)[a]	P value	Adjusted hazard or risk ratios (95% CI)[a]	Adjusted P value	PCI N = 144	No PCI N = 229	Hazard or risk ratios (95% CI)[b]	P value	Adjusted hazard or risk ratios (95% CI)[a]	Adjusted P value
**30 day outcomes**												
All-cause mortality, n (%)	4/373 (1.1%)	4/359 (1.1%)	0.95 (0.24-3.82)	0.95	-	-	1/144 (0.7%)	3/229 (1.3%)	0.53 (0.06-5.12)	0.59	-	-
Cardiovascular mortality, n (%)	3/373 (0.8%)	4/359 (1.1%)	0.72 (0.16-3.20)	0.66	-	-	1/144 (0.7%)	2/229 (0.9%)	0.80 (0.07-8.81)	0.85	-	-

© 2025 Tomii D et al. *JAMA Network Open.*

All stroke, n (%)	9/373 (2.4%)	9/359 (2.5%)	0.96 (0.38-2.41)	0.92	0.80 (0.24-2.67)	0.71	3/144 (2.1%)	6/229 (2.6%)	0.80 (0.20-3.18)	0.75	-	-
Hospitalization for valve-related dysfunction or heart failure	1/373 (0.3%)	8/359 (2.3%)	0.12 (0.01-0.95)	0.05	-	-	1/144 (0.7%)	0	-	-	-	-
Myocardial infarction, n (%)	4/373 (1.1%)	0	-	-	-	-	2/144 (1.4%)	2/229 (0.9%)	1.60 (0.22-11.34)	0.64	-	-
All bleeding, n (%)	97/373 (26.2%)	78/359 (22.0%)	1.19 (0.89-1.61)	0.25	1.09 (0.75-1.58)	0.65	44/144 (30.8%)	53/229 (23.3%)	1.39 (0.93-2.07)	0.11	1.55 (0.90-2.69)	0.12
BARC Type 2 bleeding, n (%)	30/373 (8.8%)	27/359 (8.0%)	1.07 (0.64-1.80)	0.80	0.83 (0.43-1.59)	0.57	13/144 (9.9%)	17/229 (8.1%)	1.27 (0.62-2.62)	0.51	1.64 (0.58-4.66)	0.35
BARC Type 3 or 5 bleeding, n (%)	71/373 (19.9%)	52/359 (15.3%)	1.31 (0.91-1.87)	0.14	1.23 (0.78-1.93)	0.37	33/144 (24.2%)	38/229 (17.2%)	1.44 (0.91-2.30)	0.12	1.43 (0.77-2.65)	0.26

© 2025 Tomii D et al. *JAMA Network Open*.

Values are counts of events (first occurrence per patient only, % from Kaplan-Meier estimates, Cox model with hazard ratio, and Wald test for statistical significance of the hazard ratio) for mortality, stroke, and hospitalization.

[a]Obstructive CAD vs. no obstructive CAD. [b]PCI vs. no PCI.

BARC = Bleeding Academic Research Consortium. Other abbreviations as in **Tables 1** and **2**.

© 2025 Tomii D et al. *JAMA Network Open*.

eFigure 1. Cumulative incidence of clinical outcomes according to the presence or absence of obstructive CAD

BARC = Bleeding Academic Research Consortium; CAD = coronary artery disease; CI = confidential interval; HR = hazard ratio; PCI = percutaneous coronary intervention; TAVR = transcatheter aortic valve replacement.

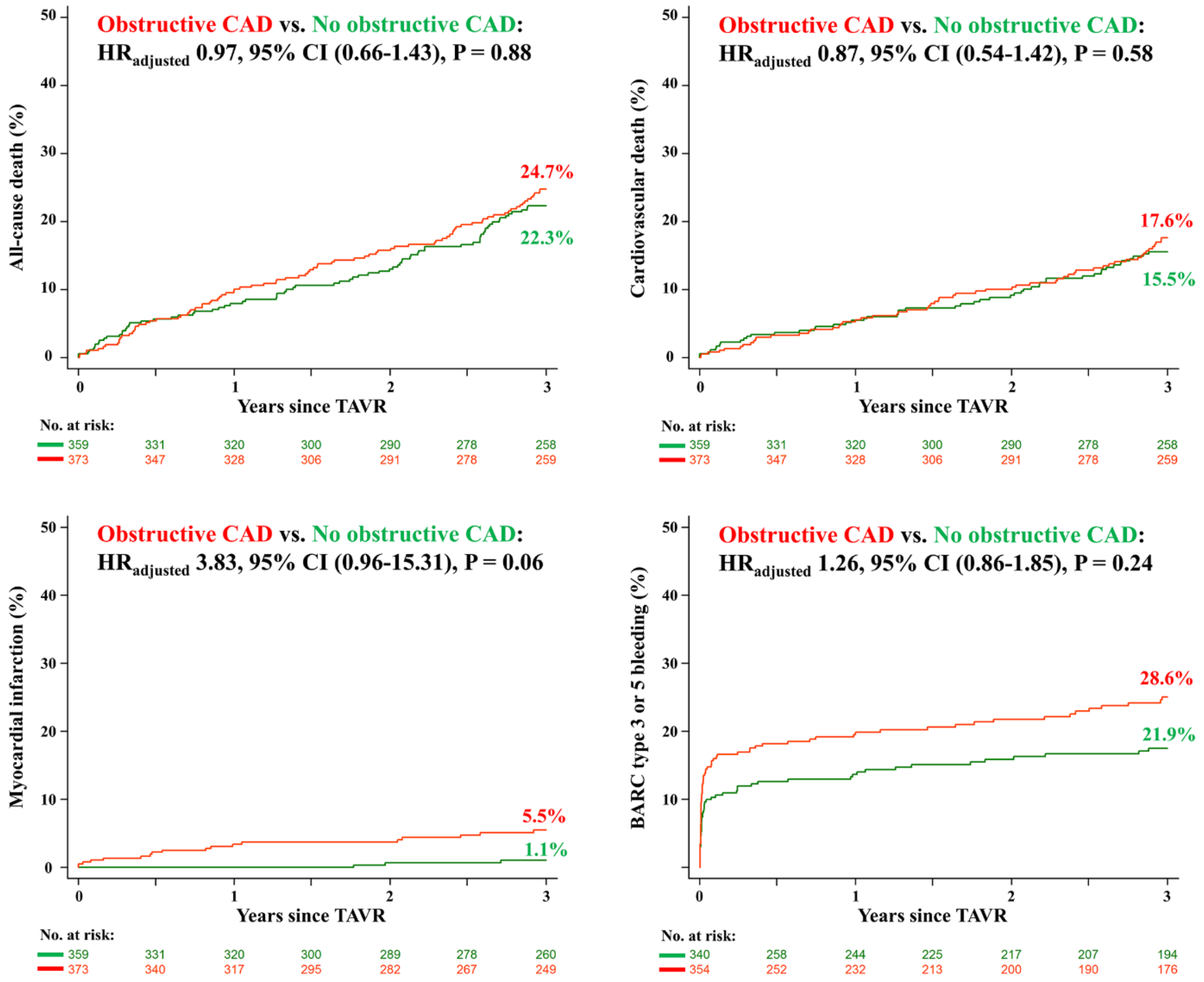

© 2025 Tomii D et al. *JAMA Network Open*.

eFigure 2. Cumulative incidence of clinical outcomes in patients with obstructive CAD according to use of PCI

BARC = Bleeding Academic Research Consortium; CAD = coronary artery disease; CI = confidential interval; HR = hazard ratio; PCI = percutaneous coronary intervention; TAVR = transcatheter aortic valve replacement.

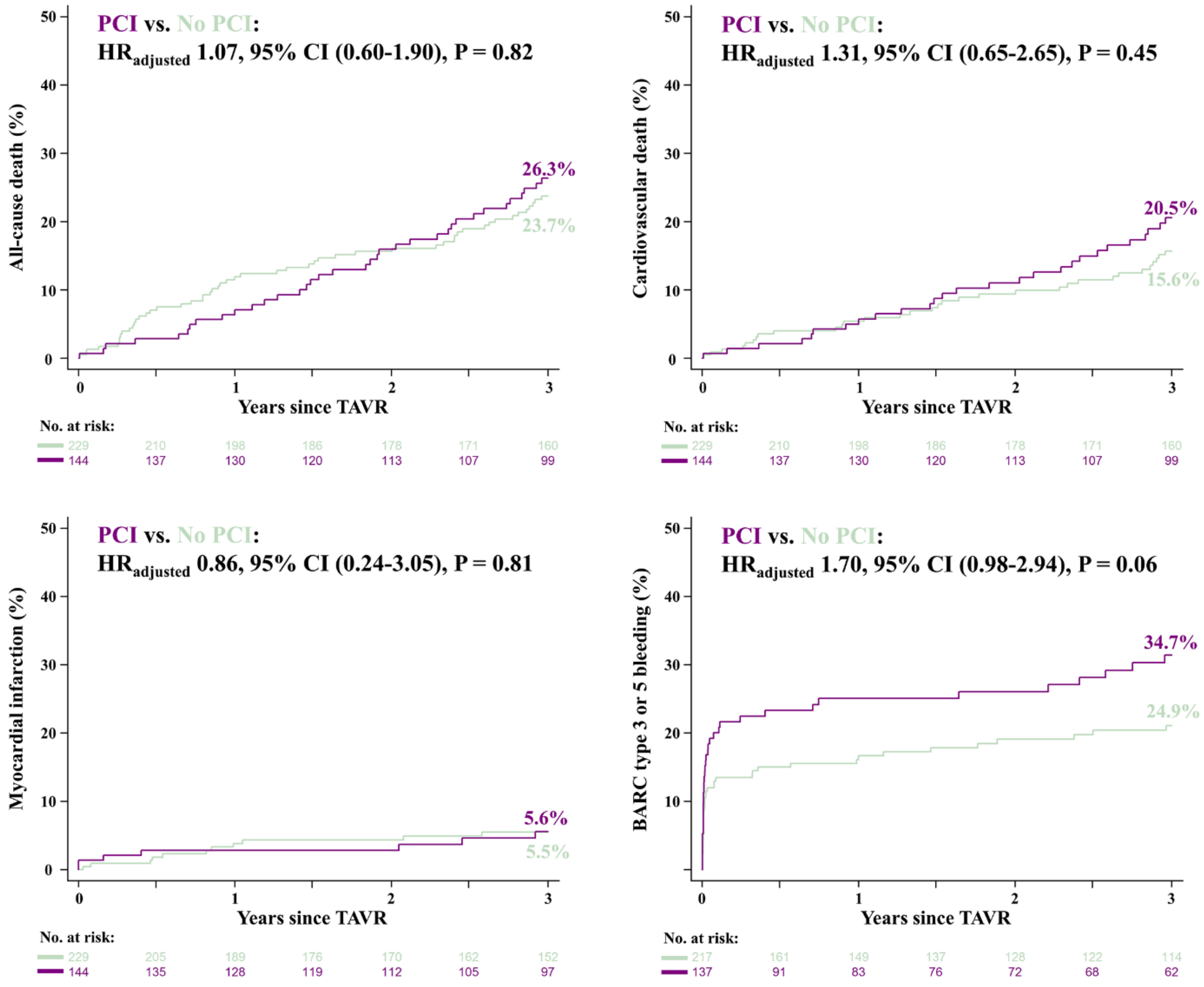

© 2025 Tomii D et al. *JAMA Network Open*.
