# Supplementary material for: Obstructive Coronary Artery Disease and Health Status in Transcatheter Aortic Valve Replacement: A Post Hoc Analysis of the SCOPE I Randomized Clinical Trial
Source: JAMA Netw Open. 2025 Dec 9;8(12):e2547111. doi: 10.1001/jamanetworkopen.2025.47111 (PMC12690430; doi:10.1001/jamanetworkopen.2025.47111)
Supplement: Supplement 2. — Data Sharing Statement [file jamanetwopen-e2547111-s002.pdf]

# Data Sharing Statement

Tomii. Obstructive Coronary Artery Disease and Health Status in Transcatheter Aortic Valve Replacement. *JAMA Netw Open*. Published December 09, 2025.
doi:10.1001/jamanetworkopen.2025.47111

**Data**
**Data available:** Yes
**Data types:** Deidentified participant data
**How to access data:** The data that support the findings of this study are available from the corresponding author upon reasonable request (thomas.pilgrim@insel.ch).
**When available:** With publication

**Supporting Documents**
**Document types:** None

**Additional Information**
**Who can access the data:** The data that support the findings of this study are available from the corresponding author upon reasonable request.
**Types of analyses:** The data will be made available for research purposes upon reasonable request and subject to approval of a methodologically sound proposal.
**Mechanisms of data availability:** Data will be made available with investigator support, after approval of a methodologically sound proposal, and under a signed data access agreement.
